# Impact of covid-19 on education, psychological wellness and life style of dental students in Saudi Arabia

**DOI:** 10.6026/97320630018588

**Published:** 2022-06-30

**Authors:** Nishath Sayed Abdul, Sarah Abdulmohsin Alarbash, Zinab Hassan Albati, Nouf Khalid Alkhelaiwi, Walaa Qasim Alkhalifa, Mahesh Shenoy

**Affiliations:** 1 Faculty of Oral Pathology, Department of OMFS and Diagnostic Sciences, College of Dentistry, Riyadh Elm University, Riyadh, Saudi Arabia; 2 Dental Students, college of Dentistry, Riyadh Elm University, Riyadh, Saudi Arabia

**Keywords:** COVID-19, dental education, psychological wellness, life style,, Saudi Arabia

## Abstract

The COVID-19 epidemic has had a significant impact on dental education, psychological health, and students' way of life worldwide. The new methods of teaching and learning had to be adapted by dental educational institutions. Due to lifestyle changes, COVID-19 had a major negative influence on students' mental health. Therefore, it is of interest to assess depression; anxiety and stress scale (DASS-21) levels experienced by dental students and compare them with gender and course of students. A cross-sectional survey based descriptive study was conducted among 356 undergraduate dental students, aged between 18-31 years, at Riyadh Elm University, Riyadh, kingdom of Saudi Arabia from January – March, 2022. A self-administered structured questionnaire written in English and Arabic language was given to all the willing participants. Four components of the questionnaire covered demographic information, the effect of COVID-19 on dental education, psychological health, and student lifestyle. In order to establish statistically significant variations across genders and student course levels regarding the effect of COVID-19 on dentistry education, the Chi-square test was used. Mann-Whitney U test was used to compare depression, anxiety, stress scales (DASS- 21) with variables such as gender and course level of students. Statistical significance was set at p-value <0.05.A total of 356 dental students were involved in this study with the total response rate of 92.2%.Majority of the respondents were males (56.2%) than females 156 (43.8), aged between 18-22 years (53.4%). Overall, moderate levels of the depression, anxiety and stress scores (DASS-21) were seen among 46.9%, 30.3% and 35.6% of the dental students, respectively. Thus, it can be concluded that COVID-19 pandemic had a profound impact on dentistry students' education, mental health, and way of life. More women and dentistry students on the clinical level felt the effects on their education. In order to implement psychological empowerment initiatives integrating institutional counseling services for students, the right steps should be done.

## Background:

The coronavirus disease 2019 (COVID-19), which first appeared on December 12th, 2019, in Wuhan, China, and caused a global pandemic of acute respiratory syndrome in people, had a significant impact on people's psychological health, education, and way of life. The COVID-19 is a highly contagious disease. At both the population and individual levels, the Saudi regime had to impose social alienation. Numerous precautions, including the closing of educational institutions, avoiding public meetings, and a nationwide lockdown, were implemented across the nation to slow the disease's rapid spread. Activities related to education and the workplaces were impacted by these abrupt closures during the COVID-19 pandemic [1]. Diverse segments of the community are currently experiencing or have had mental health concerns as a result of the COVID-19 pandemic. Despite the fact that these studies have evaluated mental health difficulties during outbreaks, the majority of them have concentrated on healthcare professionals, patients, children, and the general public. Despite the fact that many research have been done to examine students' mental health, the majority of the information came from China and Western nations [2]. Due to the pandemic, students' eating and lifestyle patterns changed. Simple carbohydrates-rich foods can lower stress levels and have a favorable impact on mood. Due to containment measures, lifestyle may be significantly altered, increasing the likelihood of sedentary behaviors and altering smoking and sleeping patterns. Additionally, nutrition also appears to have an impact on sleep quality [3]. The academic progress of students and their future professional and personal chances can both be negatively impacted by poor psychological wellbeing. There is a dearth of information on the psychological health and way of life of Saudi dentistry students during the pandemic, as well as how they have adjusted to this new teaching method. The effect of COVID-19 on dental education, psychological wellbeing, and lifestyle of dental students at a Private University is therefore of interest. Therefore, it is of interest to assess the impact of COVID-19 on dental education, psychological wellness and life style of dental students of a Private University.

## Materials and methods:

### Study design:

This is a cross-sectional, institutional-based study, which was conducted among 356 dental students, which included pre-clinical, clinical dental students and interns of Riyadh Elm University (REU), Riyadh, Saudi Arabia.

### Inclusion and exclusion criteria:

The study included those dental students who were willing to participate. An exclusion criterion included those dental students not willing to participate and those with incomplete questionnaires.

### Ethical statement:

The participants of the study were informed about the purpose and objective of the research and informed consent was obtained. Ethical clearance from the Institutional Review Board of REU with IRB approval number FRP/2021/415/651/631 was obtained.

### Data collection:

A self-administered structured questionnaire written in English and Arabic local language was distributed online among 356 dental students. The study participants included are pre-clinical level (1st year to 3rd year), clinical level dental students (4th to 6th year) and interns. Males 200 (56.2%) and females 156 (43.8%), aged between 18-31 years were included. The questions were both open-ended and close-ended type. The Questionnaire was divided into four sections. Section I consisted of demographic details of the participants, which included age gender, course level of students, marital status, accommodation and financial status. Section II assessed the impact of COVID-19 on dental education. A modified version of the questionnaire from the previous studies [4,5] was used to assess the impact of COVID-19 on dental education. Section III consisted questions on Impact of COVID-19 on psychological wellness. The psychological wellness of the dental students was assessed using the validated Arabic version of the Depression, Anxiety and Stress Scale–21 item (DASS-21) questionnaire [6] which was translated from the English version of Lovibond SH and Lovibond PF [7]. Section IV consisted of questions on the impact of COVID-19 on life style of dental students. The questionnaire for this section included 15 questions related to their eating, sleeping habits and physical activities during the pandemic.

### Pilot study and pretesting of the questionnaire:

A pilot study was conducted to test the validity and feasibility of the study. A pre-tested self -administered questionnaire, written in Arabic, local language was distributed among randomly selected 30 dental students who were willing to participate. Cronbach's coefficient was found to be 0.80, which showed internal reliability of the questionnaire.

### Data analysis:

Statistical data was analyzed using SPSS software 23.0 version (IMM Corporation, Armonk, NY, USA). Categorical responses were entered as numbers and percentages. Chi-square test was applied to find significant differences between genders, course level of students for impact of COVID-19 on dental education. Psychological wellness was assessed by variables of stress; anxiety and depression and Mann-Whitney U test was used for comparison of depression, anxiety, and stress scale (DASS- 21) with variables of gender and course level of students. Statistical significance was set at p-value <0.05.

## Results:

In the present study, a total of 386 dental students initially agreed to participate in the study. However, 30 students did not complete the questionnaire and were dropped from the final data analysis due to time constraint and unwillingness to participate. The response rate of participants was 92.2%.Majority were males 200 (56.2%) than females 156 (43.8%), aged between 18-22 years (53.4%). The majority of the participants were clinical level students (50%), single (76.1%), living with their Parents/families (48.3%). About 65.4% received financial support from their families. The demographic characteristics of the study participants were shown in (Table 1 -see PDF).

## Impact of COVID-19 on dental education of students:

A total of 39.7% female students agreed that they missed dental education experiences due to pandemic outbreak and lockdown as against 24 % of male students. This was statistically significant at p<0.05. About 76 (48.7%) of females and 43.4% of clinical level students "agreed" that online group discussion posted on learning would have a positive value on dental education than offline discussion. A total of 38.8 % clinical students agreed for online learning than offline, as compared to only 30.3 % pre -clinical and 32.1% interns. The reason for the less percentage of agreement was due to the fact that they felt less motivated and less engaged during online classes. Majority of male students 64 (32.0%) and preclinical levels (40.2%) agreed for continuation of online didactics even after the pandemic. A total of 53.6% of all course level students, with majority of clinical level students (59%) and females (62.2%) disagreed for online dental laboratory sessions. This suggests that the clinical students lacked confidence for online lab /practical training. Teachers are familiar with online mode of teaching was agreed by 52.2% clinical students and 51.8% interns. However, 32.8% pre-clinical students disagreed, which was statistically significant. Majority of females (42.3%) and interns (50%) agreed that their grades were better due to online learning than offline. Interns felt that the online teaching was not very effective with only 9(16.1%) strongly agreed as against 49 (40.2%) of preclinical students. Overall, it can be seen that female students and students with clinical exposure particularly, were not really happy with online teaching and were of the opinion that COVID-19 strongly impacted their educational performance (Table 2 - see PDF).

## Impact of COVID-19 on psychological wellness of dental students:

A significant difference in the psychological wellness of dental students was observed among different genders and course levels of students. When assessed for the psychological impact of COVID 19, it was reported that females were significantly distressed with increased levels of stress, anxiety and depression than male students. Majority of Interns (23.2%) expressed increased levels of stress than other level students due to hardships in adjusting to pandemic situation, mood changes such as agitation and irritation (Table 3 - see PDF). However, preclinical students reported elevated levels of depression and anxiety than other levels students. Female students reported increased levels of psychological stress, anxiety and depression than their male counterparts (Table 4 - see PDF). Overall, moderate levels of the depression, anxiety and stress scores (DASS-21) were seen among 46.9%, 30.3% and 35.6% of the dental students, respectively (Table 5 - see PDF).

## Impact of COVID -19 on the life style of dental students:

COVID 19 did affect the life style of dental students with many variables showed greater percentage of "yes" as compared to "no" and "don't know" in the lifestyle questionnaire (Table 6 - see PDF). About 55.8% of the dental students agreed that their life style changed with changes in their eating habits, sleeping habits social life and other physical activities due to COVID-19 pandemic. Majority (65.7%) of them agreed that they felt socially isolated and their relationship with their close friends and relatives was affected due to pandemic outbreak. The eating habits were changed in 53.4% of the students. However, sleep was not disturbed due to pandemic in 63.8% of students.

## Discussion:

### Impact of COVID -19 on Dental education:

A dental student's professional journey, which includes several stages before they are qualified to practice dentistry, is taken into account while making the switch from on-campus instruction to online learning. The behavioral and practical skills (shows and does) call for interactions in the pre-clinical and clinical contexts; the cognitive skills (knows and knows how) can be acquired and tested in a virtual environment. The online format for learning was mostly acceptable and suitable for didactic activities. Overall, 61% of the students were satisfied with online learning and majority were females (73%) in a study conducted by Coughlan et al. [8], which is similar to the present study, in which 59% of all course level students agreed for online and majority (60.2%) were females. The introduction of the online mode of dental education as against the conventional person - person interaction has presented problems such as clinical task stimulations [9] and concept of virtual computerized patients [10]. However, contradicts other studies [11, 12] where, very few numbers of students (36.1%) agreed for online mode of learning. In few other studies [11, 14] done among dental students, 83.6%, 831.3% expressed lack of confidence in their clinical /practical work, which agrees with the present study in which majority (59 %) of the students do not want online lab /practical teaching. A study by Dhanabal et al. [11] stated that majority (72.4%) agreed that their teachers were well trained for online teaching, which is similar to the present study (52.2%) of clinical level students "agreed" for teachers being familiar with online mode of teaching than others. Majority of their students (69.9%) agreed to continue in the same mode even after the pandemic [12], which contradicts our present study in which, very less that is only 31.5% of all students want to continue online learning even after the pandemic. A study conducted in Jordan [4] among dental students, 77% students agreed that they missed education experiences as a result of lock down and 66% agreed for online group discussion, This is similar to the present study, which reported that 61.5% students missed their dental educational experiences due to the outbreak of pandemic and 67.1% students agreed for group discussions. In a study by Cheng et al. [5] about 10.4% agreed for changing the dental laboratory format to online mode. This contradicts the present study, where majority (53.6%) of all students and 22.4% females disagreed for online dental laboratory format. Dentistry is a field that requires students to be skilled for providing quality dental care, which is possible only with years of rigorous training, failed to gain any clinical experiences, particularly in the lock down period will lead to stress among students which would have negative impact on dental education [13]. Majority (48.2%) of the students were females, which contradicts the present study, in which males (56.2%) were in majority than females (43.8%). About 12.6% lived alone, whereas in the present study majority (34.3%) lived alone. About 73.1% were satisfied with online learning, whereas, in the present study only 59% of all course level students were satisfied with this mode of learning. Stress was reported more among clinical level students [13], however this finding contradicts with the present study as majority were interns (23.2%).According to a study done by Farrokhi et al. [14], there were issues with teaching and learning standards. These issues included challenges with teaching students practical skills, worries about dentistry education quality assurance, online and distance learning, and other related issues (both theoretically and practically). In a study by Hassan et al. [15], majority (81.3%) of the students reported that online practical education was not effective as theoretical teaching, similar to the present study (53.6%).

### Impact of COVID -19 on psychological wellness:

Courses like dentistry and medicine, which are designed to include interaction and hands-on experiences such as clinical and practical labs. One cannot deny the fact that didactic and clinical skills are two different outcomes of education. No virtual sessions can duplicate the close experience with patients. The switch to online and those that were originally not designed for online delivery resulted in increased psychological distress among students [2]. Majority of the respondents were females (79.4%, 55.6%, 65%, 54.1%) in previous studies [2,16, 17], whereas, in our present study males (56.2%) were in majority than females (43.8%). Majority (25.7 %) stayed at home with family, similar to the present study (34.3%). Those stayed with families reported high levels of anxiety than others in these studies, which contradicts the present study, in which anxiety scores were high among those students who stayed with their friends. Studies in Saudi Arabia [17] reported depression, anxiety, and fear among dental interns. Accommodation, marital status and gender did not influence the psychological health of the participants which contradicts the present study in which females reported increased levels of depression, anxiety and stress than males and accommodation of students with their friends caused more anxiety than others. Females were more prone to depression, anxiety and stress, compared to male gender in the current study. This finding was in concordance with the studies of Stangvaltaite-M et al. [18] and Guse et al. [19]. Literature evidence showed that females were greatly impacted during the pandemic in general [20,21]. The study of Guse J et al. [18] reported higher levels of anxiety and distress in dental students as compared to their medical counterparts. Elmer et al. [22] attributed the elevated anxiety levels to sudden isolation from peers. Inability to learn and practice clinical skills, which is a necessary requirement for our profession aggravated anxiety and stress levels.

### Impact of COVID -19 on life style:

There are two major influences: staying at home (which includes digital-education, smart working, limitation of outdoors and in-gym physical activity) and stockpiling food, due to the restriction in grocery shopping. Hearing or reading continuously about COVID-19 from media can increase stress levels. Stress leads subjects toward overeating, especially 'comfort foods' rich in sugar, defined as "food craving [3]. Loss of sleep due to pandemic was reported by 32.2% of the Medical and dental students in a study at Lahore . The above statement contradicts the present study, in which majority (56.2%) of the participants reported no disturbance in sleep habit. Social distancing factors (88.3%) and younger age and female gender increased risk for stressor exposure and greater degree of stressfulness . This is similar to our current study which reported majority (65.7%) of the students felt socially isolated and younger age group between 18-31 years and females were more stressed than other participants.

### Strengths and limitations:

It the first of its kind to assess the impact of COVID 19 on dental education, psychological distress and life style changes among dental students of Saudi Arabia. However, the study has its own limitations such as convenient sampling and this study is institution-based targeted on specific population of a particular region or province and is not representative of the kingdom of entire Saudi Arabia. Therefore, further studies should be done in multiple regions or provinces, involving different populations of Saudi Arabia.

## Conclusion:

The COVID -19 pandemic caused significant impact on dental education, mental health and life style of dental students. The clinical level students and females reported that their education was affected due to the pandemic outbreak. Male students expressed low levels of psychological stress, anxiety and depression than female students. Preclinical level students reported elevated levels of anxiety and depression than clinical level students and interns.
